# Advances in Nuclear Medicine Diagnostics: The Promise of Radiolabeled Dendrimers

**DOI:** 10.3390/molecules31152709

**Published:** 2026-08-04

**Authors:** Agnieszka Maria Kołodziejczyk, Bolesław T. Karwowski

**Affiliations:** Nucleic Acids Damage Laboratory, Food Science Department, Faculty of Pharmacy, Medical University of Lodz, ul. Muszyńskiego 1, 90-151 Lodz, Poland; agnieszka.kolodziejczyk@umed.lodz.pl

**Keywords:** radiolabeled dendrimers, PAMAM dendrimers, isotope, nuclear medicine, diagnosis, SPECT, PET

## Abstract

One of the most widespread causes of mortality is cancer, and its early diagnosis is a key element of modern medicine. A crucial role is played by imaging techniques such as positron emission tomography (PET), single-photon emission computed tomography (SPECT), and computed tomography (CT), which require the use of appropriate radioactive isotope tracers to enable precise visualization of pathological changes. In recent years, hyperbranched dendrimers have attracted considerable attention due to their unique architecture and functionalization capacity, making them a promising platform for the delivery of radiotracers. The aim of this manuscript is to provide an overview of radiolabeled dendrimer conjugates, their functionalization methods, and the results of preclinical studies confirming their diagnostic potential. It pays particular attention to the stability and radiochemical purity of the systems, and their ability to overcome physiological barriers. The findings presented indicate that dendrimer-based radiotracers constitute a promising class of carriers for diagnostic imaging, although further studies are required to optimize their physicochemical and biological properties.

## 1. Introduction

The high prevalence of cancer and its significant phenotypic heterogeneity pose a major challenge for modern and future medicine, with a key role being played by nuclear medicine. This field encompasses PET and SPECT imaging techniques using appropriately selected radionuclides and radionuclide–drug conjugates; it hence serves as an integrated platform for both diagnosis and therapy [1,2]. The development of these technologies is strongly supported by various nanostructures that enable the precise delivery of radiotracers and drugs [3]. However, the design of nanoplatforms combining radioisotope imaging with therapeutic effects, and their synthesis, remain a significant challenge in modern precision nanomedicine [4]. Among the most commonly studied radiolabeled nanosystems are polymeric nanoparticles [5], liposomes [6], dendrimers [7], magnetic iron oxide nanoparticles [8], silica nanoparticles [9], carbon nanotubes [10], and inorganic metallic nanomaterials [4].

**Figure 1:**
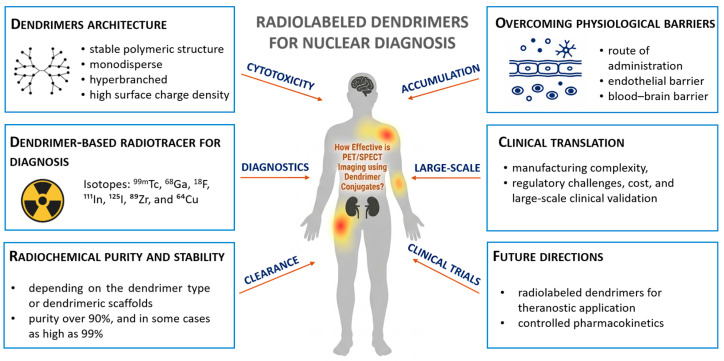
A schematic overview of the manuscript, encompassing dendrimer architecture, dendrimer-based radiotracer for PET, SPECT, *micro*-SPECT, and SPECT/CT, as well as radiochemical purity and stability; it also includes strategies for overcoming the physiological barrier and future research directions. The key topics addressed in this study include diagnostics based on radiolabeled dendrimers, their clearance, and potential accumulation.

The purpose of this manuscript is to present the current state of knowledge regarding isotope-labeled dendrimers for diagnostic application (Figure 1).

The following sections discuss (a) the architecture of dendrimers, including their hyperbranched structure, monodispersity, biodegradability, and high surface-to-mass ratio (Section 2); (b) the available dendrimer-based radiotracer, characterized by high loading capacity for radioisotopes and active substances (Section 3); (c) the radiochemical stability of the resulting systems (Section 4); and (d) the ability to overcome physiological barriers, which is crucial for the effective delivery of radiotracers to the target tissue (Section 5).

This review attempts to answer key questions regarding the development of dendrimer-based radiotracer, specifically (i) what types of dendrimers have been experimentally tested to date as platforms for radionuclides, (ii) which radioactive isotopes have been used in in vivo/ex vivo and in vitro experimental studies, and (iii) which nuclear medicine techniques and modalities have been evaluated to date in the context of these systems. The literature search was conducted in the Scopus database using a set of targeted search queries that included the following keywords: “radiolabeled dendrimers”; “dendrimers” and “radioisotopes”; and “radiolabeled dendrimers” and “clinical trials”. The review included experimental studies published between 2010 and 2026, covering, among others, the synthesis, functionalization, radioisotope labeling, and evaluation of the biological properties of dendrimers. Additionally, a supplementary search was conducted using the terms “dendrimers,” “physiological barriers,” and “radioisotopes,” which allowed for the identification of manuscripts concerning the interaction of dendrimers with physiological barriers and their pharmacokinetic and biodistribution properties. The above-mentioned search strategy allowed for obtaining a representative dataset on the current state of research on dendrimer-based radiotracers and identifying key trends and limitations in their potential application in nuclear medicine.

## 2. Dendrimer Architecture

**Figure 2:**
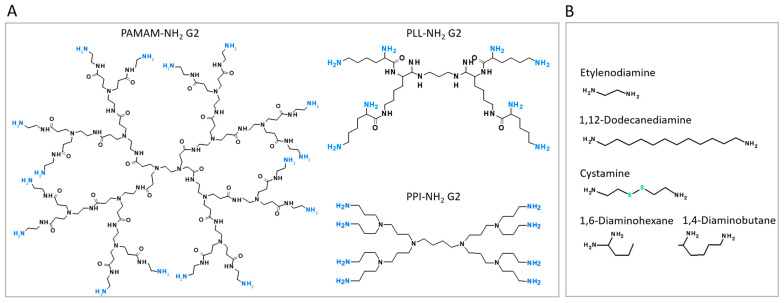
Examples of chemical structures of selected 2nd generation dendrimers, including PAMAM, PPI, and PLL, with functional amid groups highlighted in blue (**A**), and various dendrimer cores (**B**).

Dendrimers are monodisperse, stable polymeric nanostructures with a characteristic architecture composed of a core and branching units that define their generation and degree of branching [11]. The higher the generation, the more branches and functional groups there are, which can be successfully functionalized, enabling modulation of their properties, including reduced toxicity and improved biocompatibility [12,13]. However, based on our previous studies, poly(amidoamine) dendrimers functionalized with NH_2_ groups exhibit significant cytotoxicity toward human umbilical vein endothelial cells. The toxic effect is generation-dependent, and decreased cell viability is accompanied by an increased number of apoptotic cells and elevated levels of reactive oxygen species (ROS) [14,15]. The physicochemical parameters of dendrimers, such as high surface charge density [16], unique tree-like structure [11], and diversity of functional groups [17], influence their application potential in medicine and pharmacy. Among dendrimers investigated for medical applications, the most common are poly(amidoamine) (PAMAM), poly(propyleneimine) (PPI), and poly-*L*-lysine (PLL) dendrimers [7,18]. In turn, for the synthesis of radionuclide–drug dendrimer conjugates, the most commonly selected ones are PAMAM dendrimers [19,20,21], PAMAM dendrimers with entrapped AuNPs [22], PEG-citric acid linear dendrimers [23], PEGylated PAMAM dendrimers [24,25], and acid–amide dendron scaffolds. An interesting biomedical application is the use of PAMAM dendrimers as stabilizing agents for gold nanoparticles, which can serve as contrast agents in computed tomography [26,27]. Hydrophobic cores commonly used for the synthesis of PAMAM dendrimers may contain ethylenediamine, diaminododecane, diaminooxane, or diaminobutane. Figure 2 illustrates examples of chemical structures of selected dendrimers with functional groups marked in blue (A) and dendrimer cores (B).

**Figure 3:**
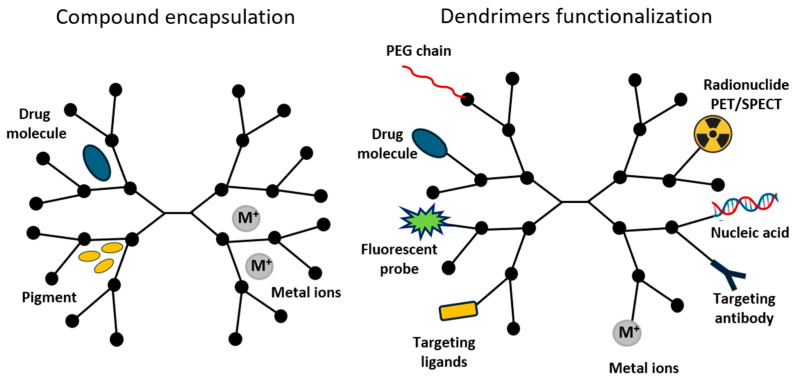
Scheme illustrating selected strategies for dendrimer compound encapsulation and functionalization. Encapsulation refers to the incorporation of substances such as drugs, metallic nanoparticles, and various fluorescent probes (pigments) within branches of dendrimers. Functionalization involves modification of functional groups and may be achieved using PEG chains, drug molecules, radioisotopes, fluorescent markers, metal ions, antibodies, or nucleic acids.

Figure 3 presents a scheme of the selected strategies for dendrimer functionalization and compound encapsulation. These modifications enable the use of dendrimers as a variety of carriers, including ligands, antibodies, fluorescent labels, and radionuclides [28]. Functional groups relevant for radionuclide applications include amide [21,25], hydroxyl [19,20,29,30], and carboxyl [31] groups.

Dendrimers occupy an intermediate position between conventional small-molecule radiotracers and larger biological targeting platforms such as peptides and antibodies [32,33]. Small molecules generally exhibit rapid tissue penetration, fast blood clearance, and high target-to-background ratios, but they often possess limited payload capacity and restricted opportunities for multifunctionalization [32]. In contrast, antibodies provide high target specificity and prolonged target retention, although their large size (typically ~150 kDa) results in slow tissue penetration, prolonged circulation, delayed imaging time points, and increased radiation exposure to non-target tissues [32]. Peptides represent an intermediate class, combining relatively rapid pharmacokinetics with good receptor specificity but limited capacity for carrying multiple imaging or therapeutic moieties [32,34]. In comparison, dendrimers offer a unique combination of multivalency, controlled architecture, and high surface functionality, enabling the simultaneous attachment of radionuclides, targeting ligands, therapeutic agents, and imaging probes within a single construct [35]. This feature permits the development of multifunctional and theranostic platforms that are difficult to achieve using conventional small molecules or peptides [35]. However, these advantages are accompanied by several limitations. Although dendrimers are often described as monodisperse macromolecules, increasing generation number and extensive surface modification may introduce variability in ligand loading and require detailed physicochemical characterization to determine the exact composition of the final product [36]. Furthermore, the relatively large hydrodynamic size of higher-generation dendrimers may hinder tissue penetration and promote accumulation within the liver, spleen, and mononuclear phagocyte system [30,36,37]. Consequently, construct size represents one of the most important design parameters affecting imaging performance [33,37]. Smaller dendrimers (e.g., G1–G4) generally exhibit faster renal elimination and lower background activity [30,36,37]. For illustrative purposes, PAMAM–NH_2_ dendrimers possess theoretical molecular weights of approximately 1.4 kDa, 3.3 kDa, 6.9 kDa, 14.2 kDa, and 28.8 kDa for generations 1 through 5, respectively [38]. Larger generations display prolonged circulation times and greater payload capacity but are often associated with increased hepatic uptake and delayed clearance [30,36,37]. Therefore, optimization of dendrimer size requires balancing multifunctionality and target retention against tissue penetration, clearance kinetics, and image contrast [33,37].

## 3. Radiolabeled Dendrimers for Diagnosis

**Figure 4:**
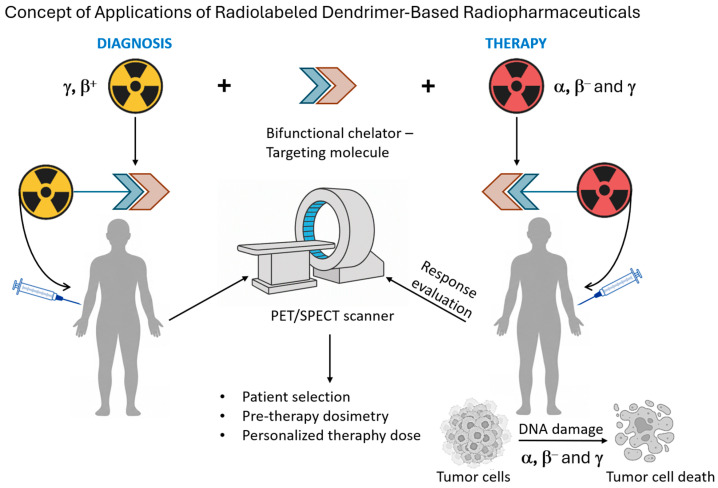
Concept of the use of radiolabeled dendrimers, highlighting their application in diagnostics and therapy, prepared based on the literature sources [40,41]. Radionuclide–drug complexes based on dendrimers consist of a targeting molecule linked to a bifunctional chelator (BFC) and tagged with gamma- or positron-emitting isotopes (γ/β^+^), which are given to the patient. Molecular imaging is then carried out with PET or SPECT to detect lesions. For treatment, the identical BFC–targeting compound labeled with a dendrimer-based radionuclide–drug is administered to the patient. Alpha, beta particles, and gamma radiation (α/β^−^ and γ) induce DNA damage, resulting in the death of tumor cells. In this situation, the assessment of the response is performed using molecular imaging.

Potential applications of dendrimer-based radiotracer-drug complexes include both precise diagnostic imaging and targeted therapeutic effects. In the context of nuclear medicine diagnostics, radiopharmaceuticals are defined as conjugated systems consisting of a radioactive isotope with biological carriers that, after administration into the body, selectively accumulate in specific tissues [7,22,39]. Figure 4 shows a diagram illustrating the potential applications of labeled dendrimers in diagnostics and therapy. In this paper, the main focus is on their diagnostic applications.

The radiation they emit can then be recorded by specialized detection systems, which enables early detection of pathological changes and assessment of their progression. Two principal imaging techniques are used in the field: positron emission tomography (PET) and single-photon emission computed tomography (SPECT) [42,43,44]. SPECT relies on γ-emitting radionuclides, whereas PET is based on positron-emitting isotopes that generate pairs of 511 keV photons as a result of annihilation events [45]. Accordingly, diagnostic radionuclides typically include γ-emitters with energies in a range of approximately 75 to 360 keV for SPECT imaging and positron-emitters for PET imaging. The use of radiopharmaceuticals at very low concentrations, in the range of 10^−6^–10^−8^ M [46], minimizes pharmacological effects while preserving their ability to provide non-invasive, real-time in vivo imaging. This enables the qualitative characterization and quantitative assessment of biological processes at the molecular and cellular levels [47,48].

Beyond qualitative visualization, PET tracers are commonly evaluated using quantitative imaging biomarkers that provide objective measures of tracer uptake and image quality. The standardized uptake value (SUV) is a commonly reported parameter and reflects the concentration of radioactivity within a tissue normalized to the injected activity and body characteristics of the subject [49]. Tumor-to-background ratio (TBR) is used to assess lesion conspicuity relative to surrounding normal tissues, whereas tumor-to-muscle ratio (TMR) compares tracer accumulation in the target lesion with that in skeletal muscle, which often serves as a reference tissue with low nonspecific uptake [49,50]. Higher SUV, TBR, and TMR values generally indicate more efficient target localization and improved imaging contrast. These metrics are particularly important when comparing different radiotracers or nanoparticle formulations because they provide complementary information to conventional biodistribution parameters, such as percentage injected dose per gram of tissue (%ID/g). Therefore, future studies on dendrimer-based PET probes should report not only tissue uptake and clearance profiles but also standardized quantitative imaging metrics to facilitate cross-study comparisons and assessment of imaging performance.

For diagnostic applications, the dendrimers have been radiolabeled with the following isotopes: ^99m^Tc [51,52], ^68^Ga [53,54,55], ^18^F [20], ^111^In [56,57], ^125^I [30], ^89^Zr [29], and ^64^Cu [30,58]. For example, dendrimers incorporating isotope-labeled gold nanoparticles have been successfully investigated for diagnostic imaging as imaging agents, particularly in SPECT and computed tomography (CT) studies [51,59].

The radionuclides investigated for dendrimer-based imaging differ considerably in their physical half-lives, decay modes, production routes, and radiation energies, which directly influence their suitability for specific nuclear medicine applications. As shown in Table 1, short-lived positron emitters such as ^18^F and ^68^Ga are particularly attractive for PET imaging, whereas ^99m^Tc and ^111^In remain widely used in SPECT-based diagnostics. In contrast, longer-lived radionuclides such as ^89^Zr enable extended imaging protocols but at the expense of higher radiation exposure. Consequently, the selection of a radionuclide for dendrimer labeling must balance imaging requirements, radiochemical considerations, and the pharmacokinetic properties of the dendrimer construct.

The design of radiolabeled dendrimers is carried out using bifunctional chelators (BFCs) [65]. Among these, one of the most widely used ligands is 1,4,7,10-tetraazacyclododecane-1,4,7,10-tetraacetic acid (DOTA). Other important chelating ligands include diethylenetriaminepentaacetic acid (DTPA), 1,4,7-triazacyclononane-1,4,7-triacetic acid (NOTA), and 1,4,8,11-tetraazacyclotetradecane-*N*,*N*,*N′*,*N″*-tetra acetic acid (TETA), which provide effective alternatives for the coordination of various metal ions [66,67]. Detailed characteristics of the preclinical studies on individual radiolabeled dendrimers in diagnostic applications are presented in the following subsections.

### 3.1. The ^99m^Tc Isotope

The ^99m^Tc isotope is widely used in diagnostic imaging, particularly in compounds such as methylenediphosphonic acid (^99m^Tc-MDP) [68] for bone scintigraphy, methoxyisobutyronitrile (^99m^Tc-MIBI) [69] for cardiac and parathyroid imaging, and diethylenetriaminepentaacetic acid (^99m^Tc-DTPA) [70] for renal scintigraphy. ^99m^Tc exhibits favorable physical properties, including a short half-life of 6.02 h (minimizing patient exposure) and the emission of gamma photons at an optimal energy (~140 keV), with minor contributions from internal conversion electrons and Auger electrons, enabling high-quality SPECT imaging at low doses [71] and radiation safety [72]. In recent years, ^99m^Tc has also been extensively applied in dendrimer-based imaging systems.

Li et al. [51] developed ^99m^Tc-labeled PAMAM G2 dendrimers with entrapped Au nanoparticles, functionalized with a CXCR4-specific ligand for targeted glioma imaging. In vitro studies conducted on HeLa-HFAR versus HeLa-LFAR cells demonstrated higher uptake of these systems in receptor-positive cells [51]. Furthermore, SPECT/CT studies performed on tumor-bearing mice confirmed targeting specificity, with greater tumor accumulation compared to non-targeted constructs. For comparison, ^68^Ga-Pentixafor, a small-molecule CXCR4 receptor ligand, is characterized by rapid blood clearance, low accumulation in most non-target tissues, and high lesion-to-background contrast [73,74]. Its favorable pharmacokinetic profile enables image acquisition as early as approximately one hour after administration, when blood-pool activity has been substantially reduced, and receptor-specific uptake predominates over nonspecific tracer distribution. Consequently, ^68^Ga-Pentixafor provides high-quality imaging and excellent visualization of CXCR4-expressing lesions [74]. On the other hand, radiotracers targeting CXCR4 that are based on dendrimers are currently at the preclinical stage of development and have been evaluated only in animal models and in vitro cell-culture systems. To the authors’ knowledge, there are no clinical studies demonstrating the superiority of dendrimer-based CXCR4 radiotracers over Pentixafor. Therefore, based on the currently available evidence, dendrimer-based CXCR4 radiotracers should be regarded as a promising experimental platform rather than an approach with a proven advantage over Pentixafor.

Subsequently, Song et al. [52] synthesized PAMAM G3 dendrimer-based systems conjugated with folic acid (FA), modified with 2-hydrazinonicotinic acid (HP3), and labeled with the ^99m^Tc isotope (^99m^Tc-HP_3_FA). These systems were evaluated both in vivo (KB tumor-bearing mice) and in vitro (KB cells), confirming cellular uptake and high tumor accumulation, with a tumor-to-muscle ratio of approximately 25.78. Strong kidney uptake was also observed due to folic acid receptor expression [52]. These results represent a promising start for ^99m^Tc-HP3FA in the early diagnosis of folic acid receptor-positive tumors [52]. In addition to lower-generation systems, higher-generation PAMAM dendrimers (e.g., G5) have also been investigated for ^99m^Tc labeling in combination with folic acid [75]. In the case of ^99m^Tc-G5-Ac-pegFA-DTPA [76], there occurred significantly higher uptake in KB cancer cells and selective accumulation in the tumor region. Additionally, uptake in KB cells depended on the incubation time [76]. For the labeled dendrimer Ac-G5-FA-1B4M DTPA, the radiochemical yield reached 98.9%, with excellent in vitro/in vivo stability, rapid blood clearance, and targeted accumulation in the tumor (*micro*-SPECT). McNelles et al. [24] used ^99m^Tc for the functionalization of high-generation aliphatic polyester dendrimers (bis MPA G5) and for effective diagnostic studies (SPECT) in rat and mouse models with H520 tumors (squamous cell carcinoma). The results obtained via SPECT confirmed the selective accumulation of radioactively labeled high-aliphatic PEGylated dendrimers in H520 tumors [24]. Xu et al. [77] studied radio-labeled PAMAM G5 dendrimer conjugates using the ^99m^Tc isotope with biotin (Bt-G5-Ac-1B4M-^99m^Tc) and avidin (Av-G5-Ac-1B4M-^99m^Tc). The primary amine groups on the surface of generation 5 poly(amidoamine) (PAMAM) dendrimers were partially acetylated in the presence of acetic anhydride and triethylamine, yielding G5-Ac dendrimers bearing an average of 81 acetyl groups per macromolecule [77]. Subsequently, G5-Ac81 was biotinylated using biotin activated with 1-[3-(dimethylamino)propyl]-3-ethylcarbodiimide hydrochloride (EDC·HCl) and 1-hydroxybenzotriazole (HOBt). The resulting intermediate was further functionalized with 2-(p-isothiocyanatobenzyl)-6-methyl-diethylenetriaminepentaacetic acid (1B4M-DTPA) to obtain the complex Bt-G5-Ac-1B4M. Finally, avidin was conjugated to the dendrimer through the high-affinity biotin–avidin interaction, affording the final construct Av-G5-Ac-1B4M [77]. Since in vitro studies on HeLa cells showed that higher uptake was achieved for the avidin conjugate, further in vitro/in vivo studies, biodistribution, and *micro*-SPECT imaging were performed for this system [77]. SPECT imaging results in nude mice with tumors confirmed the specificity of this conjugate and its potential use in further clinical trials for the diagnosis of early-stage cancer and in cancer therapy [77].

Collectively, these studies demonstrate that acetylated PAMAM and aliphatic polyester dendrimers could be effectively functionalized with the ^99m^Tc isotope for further diagnostics application. Table 2 summarizes ^99m^Tc-labeled dendrimers, including a comparison of their functionalization, biological studies, and significance.

### 3.2. The ^68^Ga Isotope

The ^68^Ga isotope is widely employed in PET imaging due to its emission of β^+^ radiation with a maximum energy of approximately 1.9 MeV (accompanied by only marginal γ emission and Auger electron emission) and a short half-life of 68 min, which enables rapid diagnosis and reduces the dose absorbed by the patient. The manuscript presents [54] a PAMAM G4–DOTA conjugate labeled with ^68^Ga, exhibiting low in vitro toxicity. Furthermore, in animal models (BALB/c mice, including the tumor-bearing Ehrlich’s ascites tumor), PET imaging revealed that these radiolabeled dendrimers accumulate in and are retained within tumor tissue—although the overall level of accumulation was low via passive/non-specific targeting, and their excretion occurs via the kidneys. Fischer et al. [53] reported acid-amine dendron scaffolds conjugated with hydrazinonicotinamide (HYNIC), an effective bifunctional chelator, which were subsequently functionalized with the ^68^Ga isotope. This radiolabeled ^68^Ga dendron scaffold has been shown to have potential utility as a multivalent PET tracer. Wangler et al. [55] reported the synthesis of various radiolabeled multimeric peptide constructs incorporating cyclic RGD pentapeptide (cRGD) fragments anchored to PAMAM dendrimer scaffolds. Systems containing multiple cRGD units exhibit more favorable properties compared to monomers and can be used, among others, for imaging the expression of αvβ3 integrin, which is present on endothelial cells in developing vasculature as well as in numerous tumor types. Multivalent binding improves retention and binding strength, which is predictive of improved tumor accumulation [55]. Table 3 presents a comprehensive overview of ^68^Ga-functionalized dendritic architectures, their chemical modification strategies, and the results of preclinical in vivo studies in animal models, together with in vitro data supporting their potential diagnostic utility.

### 3.3. The ^18^F Isotope

The ^18^F isotope is widely used in PET diagnostics due to the short range of the emitted positrons (on average 0.6–0.8 mm, up to a maximum of ~2.4 mm), which contributes to high spatial resolution of imaging. Furthermore, it is characterized by favorable β^+^ decay, leading to the emission of two annihilation photons with an energy of 511 keV, and a short half-life (110 min), which limits the dose absorbed by the patient.

The studies [19,20] have demonstrated the use of PAMAM G4 with ^18^F-labeled hydroxyl (-OH) groups (referred to as ^18^F-FMD) for potential diagnostic applications in PET. The synthesis of ^18^F-OP-801 was based on a copper-catalyzed click reaction (CuAAC) of an alkyne-functionalized hydroxyl dendrimer precursor with [^18^F]3-fluoropropyl azide in methanol [19,20]. Kuo et al. [19] presented studies on a mouse multiple sclerosis model (EAE), in which both pre-symptomatic and symptomatic inflammation of the nervous tissue was detected using ^18^F-FMD. An increased PET signal was observed in the spinal cord, correlating with disease severity. Uptake is ligand-free and based on fluid-phase endocytosis by activated myeloid cells [19]. In an LPS-induced mouse model of systemic inflammation/sepsis, Carlson et al. [20] demonstrated that brain uptake of ^18^F-FMD strongly correlated with sepsis severity. Autoradiography further confirmed regional accumulation within the central nervous system. Beyond PAMAM-based systems, ^18^F-functionalized dendritic and polymeric structures have also been explored, including linear hyperbranched amphiphilic polyglycerol polymers (hbPG) incorporating cholesterol (Ch) as a lipid-anchoring group [79]. Depending on the system architecture, distinct biodistribution profiles have been observed. Linear polymers exhibited rapid renal excretion and minimal organ retention. In contrast, for liposomes, reduced renal clearance and prolonged circulation time in the blood were noted. Notably, hbPG-based liposomes demonstrate higher blood retention compared to PEGylated counterparts, along with increased uptake by the reticuloendothelial system (RES) [79]. Table 4 presents a detailed overview of the biological models, together with an outline of the functionalization for ^18^F-labeled dendrimers.

### 3.4. The ^111^In and ^125^I Isotopes

Additional isotopes that have been investigated for complexation with dendrimers include ^111^In and ^125^I [30,56,57]. The manuscript describes [57] the synthesis of a system based on γ-polyglutamic acid (γ-PGA), employing a PAMAM G4 dendrimer labeled with ^111^In and polyethyleneimine (PEI) labeled with ^125^I. The paper also includes an evaluation of its efficacy as a melanoma-targeted imaging probe. The resulting system was shown to exhibit time-dependent uptake in B16-F10 cells, as well as accumulation in macrophage-rich organs such as the liver, spleen, and lungs. Sadekar et al. [30] labeled PAMAM-OH dendrimers of various generations (G5, G6, G7) with the radioisotope ^125^I. Biological studies were performed on a mouse model of orthotropic ovarian cancer (A2780) using iodinated PAMAM G7 dendrimers, which demonstrated the highest tumor accumulation (≈4–6% ID/g) and long-term signal retention (up to 1 week). In comparison, the HPMA copolymer showed significantly lower tumor uptake [30]. Biricová et al. [56] reported the synthesis of PAMAM G1 and G4 systems conjugated with a bifunctional DOTA analog based on a pyridine N-oxide: 10-[(4-carboxy-1-oxopyridin-2-yl)methyl]-1,4,7,10-tetraazacyclododecane-1,4,7-triacetic acid (H_4_do3a-py^NO–C^), denoted as G1-[^111^In(do3a-py^NO–C^)] and G4-[^111^In(do3a-py^NO–C^)]. Conjugation was carried out via the pyridine-4-carboxylic acid moiety, followed by radiolabelling with ^111^In. Both systems demonstrated high kinetic stability over 48 h. Biodistribution studies in a Wistar rat model revealed a more favorable profile for the G1-[^111^In(do3a-py^NO–C^)] conjugate, which was characterized by rapid elimination from the body, primarily via the renal route [56]. Overall, these studies indicate that both ^125^I and ^111^In-labeled dendrimers provide valuable platforms for imaging applications, with their biological behavior strongly dependent on dendrimer generation and structural design. Detailed uptake data for these systems are summarized in Table 5.

### 3.5. The ^89^Zr, ^3^H and ^64^Cu Isotopes

Other isotopes that have also been investigated as dendrimer conjugates include ^89^Zr [29], ^64^Cu and ^3^H [29,58,80]. ^89^Zr is a radionuclide that decays via β^+^ emission, with associated γ radiation, allowing its use in molecular imaging applications [81,82]. Due to its long half-life (~78 h), it enables the imaging of monoclonal antibodies and the assessment of tumor antigen expression in vivo. It is used in molecular diagnostics and for monitoring targeted therapies in oncology [81,82]. ^64^Cu is a PET radionuclide with multiple decay modes (β^+^ and β^−^ particles) accompanied by γ radiation and Auger electron emission, which enables its use in both diagnostics and, potentially, therapy [81]. Its half-life (~12.7 h) and favorable coordination properties make it suitable for labeling a variety of biological carriers, such as peptides, antibodies, and small molecules, for molecular imaging of tumors, angiogenesis, and metabolic processes [82].

Tritium (^3^H) is not used in classical in vivo molecular diagnostics; however, it is widely applied in in vitro and preclinical molecular studies [83,84]. Owing to its emission of low-energy β^−^ radiation and the absence of γ rays or positrons, tritium is unsuitable for detection by conventional imaging methods such as PET or SPECT. Furthermore, the very short range of β^−^ particles results in a lack of detectable signal beyond the site of decay [85]. However, in light of the potential use of tritium-labeled radioligands in pharmacological studies, as well as for assessing labeling efficiency in comparison with ^64^Cu, this work also addresses issues related to dendrimers labeled with ^3^H.

Leśniak et al. [29] conducted a comparative analysis of Gelsolin nanobody 11 (NB11) and PAMAM-OH G4 conjugated with deferoxamine (DFO). Subsequently, in PAMAM G4 conjugated with DFO, the primary amine groups were capped with butane-1,2-diol to yield G4(DFO)_3_(Bdiol)_110_. The resulting conjugates were radiolabeled with ^89^Zr and administered to mice via intra-arterial (IA) infusion into the carotid artery or intravenously (IV) via the tail vein, under conditions of either an intact blood–brain barrier (BBB) or osmotic BBB disruption (OBBBO) induced by mannitol. PET-CT imaging confirmed negligible brain accumulation of both conjugates following intravenous administration, regardless of BBB status. In contrast, intra-arterial administration of ^89^ZrNB(DFO)_3_(Bdiol)_110_ resulted in low initial brain accumulation, followed by complete clearance within one hour post-injection [29]. In the case of ^64^Cu-labeled PAMAM G5 dendrimers that were sequentially modified with fluorescein isothiocyanate (FI), FA, and 1,4,7,10-tetraazacyclododecane-1,4,7,10-tetraacetic acid (DOTA), followed by acetylation of the remaining dendrimer terminal amines, high-contrast PET imaging and tumor uptake of ~7% ID/g were achieved in a murine xenograft model [30]. In this study also observed relatively high accumulation and retention of these dendrimer systems (^64^Cu–DOTA-FA-FI G5·NHAc) in the mouse liver were also observed, which the authors explain as (i) the inescapable trapping of dendrimers by phagocytic cells, and (ii) the unfavorable demetallation of the ^64^Cu–DOTA complex in mouse liver [58]. This problem can be partially circumvented by using better chelating systems for ^64^Cu labeling, such as cross-bridged cyclam ligands or sarcophagin [86,87,88]—they could increase the stability of the metal chelate. Dendritic polyglycerol sulfate (dPGS) was also radioactively labeled with ^64^Cu or ^3^H [80]. Whilst optimizing the reaction parameters for tritium, the authors improved the radiochemical yield to over 80%, which suggests an improvement in reaction efficiency; however, for ^64^Cu labeling, the radiochemical yield was very high (over 99%) [80]. Biodistribution studies using radioactive imaging and PET conducted in healthy mice and rats showed that neutral dPG was quantitatively excreted by the kidneys, whilst polysulphated analogs accumulated mainly in the liver and spleen. Small amounts of dPGS derivatives were slowly excreted by the kidneys [80]. Beyond biodistribution, radiolabeled polymers have also been investigated for interactions with blood components. Liu et al. [89] radiolabeled hyperbranched polyglycerol (HPG–C18–PEG) with the use of ^3^H and found that it binds to red blood cell (RBC) membranes, as confirmed by confocal microscopy. Polymer concentration-dependent RBC morphological changes were achieved, including crenation/spherocytosis in saline and stomatocytosis in plasma [89]. Moreover, significant retention of radiolabeled HPG–C18–PEG suggests “hitchhiking” on RBCs and prolonged circulation potential [89]. Detailed uptake data for these systems are summarized in Table 6.

### 3.6. Current Practices of Selected Isotopes and Future Perspectives for Radiolabeled Dendrimers in Diagnosis

Table 7 summarizes the applicability of the discussed isotopes in nuclear medicine, based on the experimental results cited. A comparative analysis is presented of the use or elimination of these isotopes from common practice, together with the potential applications of radiolabeled dendrimers or dendritic structures. As described in Section 3.1, Section 3.2, Section 3.3, Section 3.4 and Section 3.5, these data are derived mainly from analyses carried out on cell lines and also include studies using animal models.

## 4. Radiochemical Yield, Purity, Stability and Specific Activity

When synthesizing dendrimer-based radiotracer, particular attention must be paid to their purity and radiochemical stability. Purity is the primary factor determining their suitability for potential testing in preclinical and subsequent clinical trials [96]. Equally important are stability and clearance pathways, as these parameters influence physiological compatibility, biodistribution, and the risk of adverse effects associated with in vivo accumulation [16].

An important but often overlooked parameter in the design of radiolabeled nanocarriers is specific activity (A_s_) or molar activity (A_m_) [97]. A_m_, expressed in Bq/mol, denotes the amount of radioactivity per mole of a compound and is used when the molecular weight of the labeled substance is known, whereas A_s_, expressed in Bq/g, is typically applied to macromolecules such as proteins and antibodies when the molecular weight of the labeled material is unknown [97]. In molecular imaging, particularly when targeting receptors or biomarkers expressed at low levels, high A_s_ is desirable because it reduces the amount of non-radioactive carrier administered together with the radiotracer. Excess unlabeled molecules may compete with the radiolabeled fraction for binding sites, potentially leading to receptor saturation and reduced target uptake [97,98]. This consideration is particularly relevant for dendrimer-based radiopharmaceuticals, as their highly branched architecture enables the attachment of multiple chelating groups and radionuclides. Optimizing A_s_ may therefore improve imaging sensitivity, target-to-background ratios, and the detection of low-abundance molecular targets while maintaining favorable pharmacokinetic properties [98]. The importance of molar activity is particularly evident in receptor-targeted dendrimer systems described in this review. For example, Fischer et al. [53] reported acid–amide dendron scaffolds radiolabeled with ^68^Ga that achieved specific activities of up to approximately 163 GBq/µmol while maintaining high radiochemical purity (>95%). Such values are advantageous for molecular imaging because they enable administration of a lower mass of carrier while preserving sufficient signal intensity. This consideration becomes especially relevant for radiolabeled dendrimers targeting receptors expressed at low or moderate densities, such as folate receptors, CXCR4, or integrins, where excess unlabeled dendrimer could potentially compete with the radiolabeled fraction for available binding sites [51]. Moreover, the multivalent architecture of dendrimers enables the incorporation of multiple chelators and radionuclides within a single nanostructure, providing opportunities to enhance the effective activity delivered to the target tissue while maintaining favorable target-to-background ratios [54,55]. Therefore, optimization of molar activity should be considered alongside radiochemical purity and stability as a key parameter during the design of dendrimer-based radiopharmaceuticals for molecular imaging.

The nanoprobes described in [51], (Au0)6–G2–^99m^Tc–DTPA–(PEG–FC131), exhibit high radiochemical stability and excellent radiochemical purity (RP). Similarly, acid–amide dendron scaffolds functionalized with ^68^Ga have been reported to demonstrate high RP (≥95%) and high specific activity (up to ~163 GBq/µmol) [53]. Additionally, the study presented in the manuscript [20] showed that the ^18^F-FMD product achieved RP exceeding 95% along with high plasma stability. Furthermore, Fischer et al. [53] reported that acid–amine dendron scaffolds conjugated with HYNIC also exhibited high RP (≥95%). Comparable results have been observed for other radionuclides. Orocio-Rodriguez et al. [99] reported that the RP for both nanosized conjugates ^99m^Tc-PAMAM-Tyr3-octreotide and ^99m^Tc-AuNP-Tyr3-octreotide was above 94%. Lesniak et al. [29] reported that both the dendrimer and the Gelsolin nanobody 11 radiolabeled with ^89^Zr exhibited a radiochemical purity of 99%. The radiochemical purity of the aliphatic polyester dendrimers (bis MPA G5) radiolabeled with ^99m^Tc [24] was over 99%. Additionally, Tanaka et al. [57] investigated radiochemical purities of ^111^In-DTPA-G4, ^125^I-PEI, and ^111^In-DTPA-G4/125I-PEI/γ-PGA, which were greater than 90% for both ^111^In and ^125^I. Zhang et al. [75] determined that all the conjugates, including ^99m^Tc-Ac-G5-pegFA-DTPA, ^99m^Tc-Ac-G5-FA-DTPA, and ^99m^Tc-Ac-G5-DTPA, gave excellent radiochemical purity, i.e., above 95%. For the labeled dendrimer Ac-G5-FA-1B4M DTPA, the radiochemical yield reached 98.9%, with excellent in vitro/in vivo stability, rapid blood clearance, and targeted accumulation in the tumor [78]. Overall, the majority of reported dendrimer-based radioconjugates exhibit radiochemical purities exceeding 90%, frequently approaching or surpassing 99%. Such high purity reflects the efficiency of labeling methodologies and the stability of the resulting constructs. This is of significant importance for their further use, as it minimizes the presence of undesirable byproducts that could affect the compound’s bioavailability, pharmacokinetics, and safety profile. Collectively, these findings support the continued development of radiolabeled dendrimers for diagnostic and therapeutic applications, particularly in the context of preclinical evaluation and future clinical translation.

## 5. Overcoming Physiological Barriers

The targeting of radiopharmaceuticals requires not only an analysis of their diagnostic functions, but also consideration of clinical indications and the appropriate route of administration [42,100]. Although most radiopharmaceuticals used in nuclear medicine are administered intravenously, other routes of administration are also available, such as inhaled (e.g., in lung ventilation procedures), oral (e.g., in the assessment of gastrointestinal transit or the diagnosis of gastrointestinal bleeding), and intradermal (e.g., in sentinel lymph node identification procedures) [101,102]. A significant challenge for radioisotope-labeled dendrimers is the need to overcome a few physiological barriers in order to reach the target tissues [103]. In the case of intravenous administration, the following factors are of key importance: the endothelial barrier, interactions with serum proteins, shear stress in the circulation, interactions with blood cells, and the overall stability of the system [104]. The choice of an imaging method and target site determines the requirements for the carrier’s properties; however, the time taken to achieve optimal contrast and the kinetics of elimination of radiolabeled dendrimers from the body remain equally important [7]. Another significant aspect is the assessment of the processes related to the clearance of these nanostructures, as well as the potential risks arising from their accumulation in an organ, including the possibility of inducing inflammatory reactions or neoplastic changes [105]. The ability of dendrimer-based imaging probes to overcome physiological barriers, particularly the blood–brain barrier (BBB), is strongly influenced by their structural design. Several parameters play critical roles, including particle size, surface charge, terminal functional groups, hydrophilicity, and the presence of targeting ligands [106]. In general, cationic dendrimers exhibit strong electrostatic interactions with negatively charged cell membranes and plasma proteins, which may enhance cellular uptake but can also increase nonspecific binding, toxicity, and clearance by the mononuclear phagocyte system [16]. In contrast, neutral or hydroxyl-terminated dendrimers display reduced protein adsorption and improved biocompatibility, resulting in more favorable pharmacokinetic profiles and prolonged circulation times [16,17,106]. Surface engineering through PEGylation further reduces opsonization and uptake by the reticuloendothelial system, thereby improving systemic distribution and increasing the probability of reaching target tissues [17]. Dendrimer size is another important determinant, since lower-generation structures are generally more susceptible to renal elimination, whereas larger dendrimers tend to exhibit prolonged circulation accompanied by increased accumulation in the liver and spleen [24,30].

These design principles help explain the distinct biological behavior observed among the radiolabeled dendrimers discussed in this review. For example, the avidin-modified PAMAM G5 conjugate (Av-G5-Ac-1B4M-^99m^Tc) displayed negligible brain uptake following intravenous administration, suggesting limited BBB penetration despite efficient cellular targeting in peripheral tissues [77]. In contrast, the hydroxyl-terminated PAMAM-OH G4 tracer ^18^F-flurimedrimer (^18^F-FMD, previously ^18^F-OP-801) demonstrated accumulation in regions with a compromised BBB and selective uptake by activated myeloid cells through fluid-phase endocytosis [19,20]. Similarly, Lesniak et al. reported that near-neutral PAMAM-OH G4 dendrimers functionalized with DFO and labeled with ^89^Zr showed negligible brain accumulation after intravenous administration, indicating that surface neutrality alone is insufficient for transport across an intact BBB [29]. Collectively, these findings suggest that successful access to the central nervous system depends not on a single physicochemical parameter but on the interplay between dendrimer size, surface chemistry, charge, and local BBB integrity. Consequently, future generations of radiolabeled dendrimers intended for neuroimaging should be designed by combining optimized surface properties with active targeting strategies capable of promoting receptor-mediated transcytosis across the BBB [106].

## 6. Major Barriers to Clinical Translation

Scaling up the production of dendrimer-based radiotracers from laboratory to industrial scale is a complex process due to the limited availability of the intermediates used in the synthesis, the need to ensure appropriate technological infrastructure, and the high costs and production times involved [107]. An additional challenge is posed by purification methods commonly used at the laboratory stage, such as ultrafiltration and evaporation. In large-scale production, their efficiency may be limited, which increases the risk of organic solvent residues being present in the final product [107,108]. When scaling up production, it is also necessary to assess the reproducibility of the physicochemical and biological properties of the resulting preparations. Potential variations may arise even from slight deviations between batches of raw materials used in the synthesis of nanocarriers [109]. To date, dendrimer-based radiotracers have been evaluated mainly in preclinical animal models [19,20,22,24,52], which only partially reflect the course of diseases occurring in humans. However, the use of such models is essential for obtaining reproducible results relating to specific disease states, rather than solely to the characteristics of a given animal model. Despite their limited ability to reflect the full spectrum of clinical disease, animal models provide valuable data for assessing the potential efficacy and safety of new therapeutic approaches in selected patient groups. However, anatomical and physiological differences between animals and humans must be taken into account, as these may affect the bioavailability, distribution and efficacy of medicinal products, particularly depending on the route of administration. From a biological perspective, it is also crucial to understand the behavior of nanocarriers in vivo, including their mechanisms of action and their interactions with blood, tissues, cells and intracellular compartments of the body, under both physiological and pathological conditions [110,111]. Furthermore, preclinical studies should be conducted in accordance with the principles of randomization and blinding, which help to minimize the risk of systematic error. It is also essential to include appropriate control groups, including treatments that represent the current standard of clinical care. Other important aspects relating to the implementation of industrial-scale production include intellectual property issues, regulatory requirements and an analysis of cost-effectiveness compared with currently used diagnostic and therapeutic methods [107,108,109]. It will also be necessary to develop procedures in accordance with the principles of Good Manufacturing Practice (GMP) for dendrimer-based radiotracers and to establish appropriate quality control standards, enabling their parameters to be compared with those of other radiotracers used in nuclear medicine. In the longer term, it will also be necessary to develop optimal dosing regimens and to evaluate various combination therapy strategies, supported by the results of clinical trials and medical indications.

## 7. Conclusions

Due to their high charge-carrying capacity, controlled structure and ability to be conjugated with radionuclides and directing ligands, dendrimers represent a promising diagnostic platform in nuclear medicine. The regulation of size, surface charge and degree of branching translates into the predicted biodistribution and clearance profiles of radiolabeled dendrimers. The obtained conclusions are outlined in the following points:

- The high density of dendrimers’ functional groups enables simultaneous labeling with multiple molecules, which increases imaging sensitivity and allows for the creation of multimodal systems.
- Functionalization of radiolabeled dendrimers with antibodies or peptides ensures precise targeting of specific cell populations, which can enhance imaging contrast and improve the signal-to-background ratio.
- Their favorable radiochemical stability properties, which depend, among others, on the chelator used and are crucial for maintaining the integrity of the radionuclide–carrier complex under physiological conditions. However, in the case of some isotopes (e.g., ^64^Cu), it may be necessary to conduct studies using other chelating systems that could enhance the stability of the metal chelate and also increase radiochemical yield.
- The high radiochemical purity of these radiolabeled dendrimers, which is a characteristic feature of such systems, is a key requirement for diagnostic applications in nuclear medicine, where precise quality control of the preparation is essential.
- A limitation is the potential retention in the liver and kidneys, typical of nanomaterials, which requires further optimization of the dendrimer surface (PEGylation, charge modification).

Radiolabeled dendrimers can serve as theranostic platforms, combining diagnostics with the ability to deliver drugs or therapeutic isotopes (e.g., diagnostic-therapeutic pairs), which is in line with the concept of precision medicine. Their development meets the growing demand for carriers with high specificity and controlled pharmacokinetics, which can improve the quality of imaging and enable earlier detection of pathological changes.

However, it should be noted that the process of developing a radiolabeled compound for use in nuclear medicine is a multi-stage one, and in addition to the factors mentioned above—such as synthesis optimization, stability, yield and purity—there are other important issues to consider, including large-scale production, costs, and ‘fresh’ synthesis due to the short half-life. Subsequently, the next stages involve regulatory requirements from synthesis to implementation, clinical trials, registration and implementation, clinical production and distribution.

## Figures and Tables

**Table 1:** Comparison of radionuclides investigated for dendrimer-based molecular imaging based on [60,61,62,63,64].

Radionuclide	Half-Life	Mode of Production	Mode of Decay %	Energy Emitter	References
^99m^Tc	6.02 h	^99^Mo/^99m^Tc generator	IT (100)	140 keV	[61]
^18^F	109.7 min	Cyclotron	β^+^ (97), EC (3)	635 keV E_max_	[62]
^68^Ga	67.8 min	^68^Ge/^68^Ga generator	β^+^ (90), EC (10)	1.9 keV E_max_	[60]
^89^Zr	78.4 h	Cyclotron	β^+^ (100)	245 keV E_max_	[60]
^3^H	12.3 years	Cyclotron	β^−^ (100)	18.6 keV	[63]
^64^Cu	12.7 h	Nuclear reactor	β^−^ (40), EC (40), β^+^ (19)	β^+^ 655 keV E_max_, 278 keV E_max_	[60]
^125^I	59.4 d	Cyclotron	EC (100)	186 keV	[64]
^111^In	2.83 d	Cyclotron	EC (100)	245 keV	[60]

**Table 2:** Summary of ^99m^Tc-radiolabeled dendrimers for potential diagnostic application. Dendrimer type, functionalization steps, and biological models were included. The diagnostic applicability was assessed via SPECT, SPECT/CT, or *micro*-SPECT imaging.

Dendrimer Type and/or Product Name	Functionalization	Biological Model and Findings	Ref.
PAMAM G2 with entrapped Au NP (Au DENPs)	(Au0)_6_-PAMAM G2-^99m^Tc-DTPA-(PEG-FC131): DTPA chelator + PEG spacer + FC131 (targeting ligand for CXCR4—glioma imaging).	HeLa-HFAR vs. HeLa-LFAR cells: ~3.5× higher uptake in receptor-positive cells. Tumor-bearing mice: effective SPECT/CT imaging with targeting specificity.	[51]
PAMAM G3 (~1.27 nm)	^99^ᵐTc-HP3FA: folic acid conjugation (~6–8 per dendrimer) and HYNIC-based ^99^ᵐTc labeling (tricine/TPPTS).	KB (FR+) vs. 7721 (FR−): uptake ~27% at 1 h in KB cells, blocked by FA. KB tumor-bearing mice, high tumor accumulation.	[52]
PAMAM G5 (acetylated; FA-PEG or FA conjugated; DTPA-modified)	Partial acetylation (≈81 acetamide groups) to reduce toxicity; biotinylation (~9.5 residues); conjugation with 1B4M-DTPA chelator; avidin binding via biotin–avidin interaction.	KB cells (FR+ and FR−)—PEGylated FA conjugate shows highest uptake (~15% at 6 h). KB tumor-bearing nude mice: rapid blood clearance; high kidney uptake (~18–28% ID/g), significant liver accumulation (~22–32% ID/g), tumor accumulation increased over time.	[75]
PAMAM G5 (acetylated, FA-conjugated, DTPA-chelated)	Partial acetylation (~77 groups) to reduce nonspecific interactions; folic acid conjugation (γ-carboxyl, FR targeting); 1B4M-DTPA attachment.	KB tumor-bearing nude mice: rapid blood clearance (t_1/2_α ~13.5 min); significant tumor accumulation increasing over time (~13.3% ID/g at 6 h), high kidney uptake (FR-mediated) and moderate liver uptake (~9.5% ID/g).	[78]
High-generation aliphatic polyester dendrimer (bis MPA G5) PEGylated	Core: dipicolylamine (DPA) chelator for ^99^ᵐTc(CO)_3_; periphery: vinyl groups → thiolene click PEGylation (mPEG 750).	Rats and H520 tumor-bearing mice: long blood circulation up to 24 h; primarily blood pool → later liver/spleen clearance.	[24]
PAMAM G5 (partially acetylated) conjugated with biotin, DTPA (1B4M), and avidin (Av G5-Ac-1B4M)	Partial acetylation (~81 groups) to reduce toxicity; biotinylation (~9.5 residues); 1B4M-DTPA conjugation; avidin binding via biotin–avidin interaction.	HeLa cells: high uptake (~56% at 8 h) for avidin conjugate vs. <4% for control. Kunming mice: rapid blood clearance (t_1/2_α ≈ 1.1 min, t_1/2_β ≈ 89.9 min); high accumulation in liver (~45–56% ID/g) and spleen (~43–51% ID/g), moderate lung uptake.	[77]

Folate receptor (FR), folic acid (FA), diethylenetriaminepentaacetic acid (DTPA), polyethylene glycol (PEG), dipicolylamine (DPA), percent of injected dose per gram of tissue (ID/g), 2-(p-isothiocyanatobenzyl)-6-methyl-diethylenetriaminepentaacetic acid (1B4M-DTPA), half-life of the radiolabeled conjugate (t_1/2_α), clear-phase half-life (t_1/2_β).

**Table 3:** Summary of ^68^Ga-radiolabeled dendrimers for potential diagnostics application. Dendrimer type, functionalization steps, and biological models were included. The diagnostic applicability was assessed via PET imaging.

Dendrimer Type and/or Product Name	Functionalization	Biological Model and Findings	Ref.
PAMAM G4–DOTA	Conjugation of multiple DOTA–NHS (~48 chelators/dendrimer) to PAMAM G4	BALB/c mice (tumor-bearing EAT model): rapid blood clearance (>50% in 30 min, <1% at 1 h); predominant renal excretion; low tumor accumulation (~0.33% ID/g at 1 h).	[54]
Acid–amide dendrons (solid-phase synthesis, up to 16 functional groups; DOTA-functionalized)	Solid-phase Fmoc dendrons with OEG spacers; terminal maleimide for thiol-click; focal DOTA; biomolecule multimerization.	Applicability for multivalent PET tracers; high specific activity (up to ~163 GBq/µmol); improved binding potential due to multivalency	[53]
PAMAM dendrimer scaffold (multivalent cRGD peptide conjugates, up to hexadecimer)	Functionalization with cRGD (1–16 peptides; maleimide–thiol most efficient); DOTA–PEG chelator; multimerization enhances avidity.	In vitro αvβ3 binding (U87MG): avidity increases with cRGD number (up to ~100–125× vs. monomer).	[55]

1,4,7,10-tetraazacyclododecane-1,4,7,10-tetraacetic acid (DOTA), arginine–glycine–aspartic acid (RGD), Ehrlich’s ascites tumor (EAT), percent of injected dose per gram of tissue (ID/g).

**Table 4:** Summary of ^18^F-radiolabeled dendrimers for potential diagnostics application. Dendrimer type, functionalization steps, and biological models were included. The diagnostic applicability was assessed via PET imaging.

Dendrimer Type and/or Product Name	Functionalization	Biological Model and Findings	Ref.
PAMAM-OH G4; ^18^F-flurimedrimer/^18^F-FMD, previously ^18^F-OP-801	Radiolabeling via ^18^F-3-fluoropropyl azide and CuAAC click reaction with PAMAM-G4-OH-alkyne_10_.	Mouse EAE model: detection of pre- and symptomatic neuroinflammation; uptake in activated microglia and infiltrating immune cells.	[19]
PAMAM-OH G4; ^18^F-OP-801	Labeling through ^18^F-3-fluoropropyl azide and CuAAC click reaction with an alkyne-functionalized dendrimer precursor.	LPS-induced systemic inflammation/sepsis mouse model. Uptake: inflamed brain, liver, lung, spleen and intestines.	[20]
Linear PEG and linear hyperbranched polyglycerol (hbPG) cholesterol-anchored dendritic-like polymer lipids	Cholesterol anchor with polymer (PEG or PEG-hbPG); terminal alkyne enables CuAAC labeling with ^18^F-azide; incorporated into liposomes (DOPC/cholesterol/polymer)	Free polymers: rapid renal clearance, minimal retention; liposomes: reduced clearance and prolonged circulation; hbPG-liposomes: higher blood retention but increased RES uptake.	[79]

Polyethylene glycol (PEG), reticuloendothelial system (RES).

**Table 5:** The summary of ^111^In and ^125^I-radiolabeled dendrimers for potential diagnostics application. Dendrimer type, functionalization steps, and biological models were included.

Dendrimer Type and/or Product Name	Isotope Type	Functionalization	Biological Model and Findings	Ref.
Self-assembled γ-PGA ternary nanoparticle with PAMAM G4; ^111^In-DTPA-G4/^125^I-PEI/γ-PGA	^111^In + ^125^I	PAMAM G4 conjugated with DTPA and labeled with ^111^In; combined electrostatically with ^125^I-labeled PEI and γ-PGA	B16-F10 melanoma model (cells, normal and metastatic mice): lung accumulation increases in metastasis (33.7 vs. 25.1% ID/g), with higher lung-to-blood ratios; reduced by excess γ-PGA, indicating γ-PGA-mediated targeting.	[57]
PAMAM-OH G5, G6, G7 vs. linear HPMA copolymers	^125^I	Radiolabeling with ^125^I via Bolton–Hunter reagent; HPMA radiolabeled via iodination of tyrosine groups; no targeting ligands	A2780 ovarian tumor model: G5-OH accumulates in kidneys (~150% ID/g); G6-OH in the liver and the kidneys; G7-OH shows longest circulation and highest tumor uptake (≈4–6% ID/g). HPMA 26 kDa clears rapidly; 52 kDa shows moderate circulation and low tumor uptake.	[30]
PAMAM G1 and G4	^111^In	PAMAM (G1, G4) conjugated with a DOTA-type chelator (H_4_do3a-py^NO−C^), enabling stable O_4_N_4_ coordination.	Wistar rats: G1 clears rapidly with renal excretion; G4 shows prolonged circulation and accumulates in the liver (~20.6% ID at 48 h) and the kidneys (~5.85% ID at 48 h).	[56]

Percent of injected dose per gram of tissue (ID/g), γ-polyglutamic acid (γ-PGA), poly(N-(2-hydroxypropyl)methacrylamide) (HPMA).

**Table 6:** The summary of ^89^Zr, ^64^Cu, and ^3^H for dendrimer radiolabeling. Dendrimer type, functionalization steps, and biological models were included.

Dendrimer Type and/or Product Name	Isotope Type	Functionalization	Biological Model and Findings	Ref.
PAMAM-OH G4; ^89^Zr-G4(DFO)_3_(Bdiol)_110_, compared with similarly sized nanobody	^89^Zr	G4 PAMAM-NH_2_ conjugated with ~3 DFO chelators, then remaining amines capped with butane-1,2-diol to yield near-neutral hydroxyl-like surface	Healthy C3HeB/FeJ mice: IV dendrimer shows negligible brain uptake; IA dendrimer yields low, transient signal (~3.2–3.3% ID/cc), clearing within 1 h; IA nanobody shows higher uptake and partial 24 h retention.	[29]
PAMAM G5	^64^Cu	DOTA chelator + FA + FI + acetylation of terminal amines	KB (FR+) vs. A549 (FR−) cells, selective uptake and blocking with FA. Murine xenograft: tumor uptake ~7% ID/g, high contrast PET imaging.	[58]
Dendritic polyglycerol (dPG) and sulfated dPG (dPGS, ~10 kDa).	^64^Cu or ^3^H	^64^Cu: conjugation of DMPTACN chelators (isothiocyanate or maleimide) to amino-functionalized dPGS followed by radiometal complexation	Mouse and rats: neutral dPG shows rapid renal clearance (~50% at 1 h); dPGS accumulates in the liver and spleen (RES) with long retention (up to 60% at 10 days, ~40% at 30 days).	[80]
Hyperbranched, amphiphilic polyglycerol (HPG–C18–PEG)	^3^H	Hyperbranched polyglycerol with C18 stearoyl and PEG chains; ^3^H-labeled for adsorption and FITC-labeled for localization.	RBCs: strong membrane adsorption via C18 chains; Langmuir-type (up to ~10^6^ molecules/cell; ~10^4^ after plasma washing).	[89]

Dendritic polyglycerol sulfate (dPGS), human red blood cells (RBCs), deferoxamine (DFO), folic acid (FA), fluorescein isothiocyanate (FI), intra-arterial (IA) infusion, intravenously (IV) injection, dimethyl-substituted 1,4,7-triazacyclononane (DMPTACN).

**Table 7:** Overview of selected radionuclides used in molecular imaging, including their imaging modalities, current diagnostic applications, and prospective use of dendrimer-based isotope conjugates for future diagnostics.

Isotope	Type of Diagnostic Imaging/Radiation	Recent Use—Diagnostic Model	Applicability of Dendrimer-Based Isotope Conjugates—Future Diagnostic
^99m^Tc	SPECT/SPECT-CT γ radiation, Auger electrons	scintigraphy of bone metastases, cardiac perfusion, thyroid, renal, liver, lung (ventilation/perfusion scans), and hepatobiliary imaging [90]	cervical cancer [52], lung cancer [24]
^68^Ga	PET/β^+^ particles, γ radiation, Auger electron	neuroendocrine tumors, prostate cancer [91]	mammary adenocarcinoma [55], glioblastoma [55]
^18^F	PET/β^+^ particles, γ radiation	lung, breast, colorectal, head and neck cancers, lymphoma and melanoma [92]	multiple sclerosis [19], systemic inflammation/sepsis [20]
^111^In	SPECT/γ radiation, Auger electrons	previously used—neuroendocrine tumor, replaced by ^68^Ga [93,94]	melanoma [57]
^125^I	SPECT/γ radiation, Auger electrons	past used—prostate cancer, largely replaced by ^99m^Tc, ^18^F, ^68^Ga [95]	ovarian tumor [30]
^89^Zr	PET/β^+^ emission, with associated γ radiation	depending on the antibody used: breast, colorectal, prostate, lung cancers [81,82]	neurodegenerative disorders [29]
^3^H	β^−^ particles	not commonly used in clinical medicine, biomedical sciences, and pharmacology tests [83]	molecular labeling [89]
^64^Cu	PET/β^+^ and β^−^ particles, accompanied by γ radiation and Auger electrons	depending on the molecule used: breast, prostate, neuroendocrine, colorectal cancers and glioblastoma [82]	cervical cancer [58]

## Data Availability

No new data were created or analyzed in this study. Data sharing is not applicable to this article.

## Abbreviations

The following abbreviations are used in this manuscript:

PAMAM	poly(amidoamine)
POPAM	poly(propyleneimine)
PLL	poly-*L*-lysine
TETA	1,4,8,11-tetraazacyclotetradecane-*N*,*N*,*N′*,*N″*-tetraacetic acid
NOTA	1,4,7-triazacyclononane-1,4,7-triacetic acid
BFC	bifunctional chelators
PET	positron emission tomography
SPECT	single-photon emission computed tomography
CT	computed tomography
MRI	magnetic resonance imaging
PEG	polyethylene glycol
DTPA	diethylenetriaminepentaacetic acid
LET	linear energy transfer
DOTA	1,4,7,10-tetraazacyclododecane-1,4,7,10-tetraacetic acid
HYNIC	hydrazinonicotinamide
FR	folate receptor
